# Healthcare Workers’ Perceptions on the “SaferBirths Bundle of Care”: A Qualitative Study

**DOI:** 10.3390/healthcare11111589

**Published:** 2023-05-29

**Authors:** Paschal Mdoe, Estomih Mduma, Sara Rivenes Lafontan, Hege Ersdal, Catherine Massay, Vickfarajaeli Daudi, Damas Kayera, Shally Mwashemela, Robert Moshiro, Benjamin Kamala

**Affiliations:** 1 Haydom Lutheran Hospital, Haydom 9000, Tanzania; estomduma@gmail.com (E.M.);; 2 Faculty of Health Sciences, Oslo Metropolitan University, 0167 Oslo, Norway; 3 Department of Simulation, Stavanger University Hospital, 4068 Stavanger, Norway; 4 Faculty of Health Sciences, University of Stavanger, 4021 Stavanger, Norway; 5 Kilimanjaro Christian Medical University College, Tumaini University Makumira, Moshi 25102, Tanzania; 6 UNICEF, Dar es Salaam 4076, Tanzania; 7 Department of Pediatrics, Muhimbili National Hospital, Dar es Salaam 65000, Tanzania; 8 School of Public Health and Social Sciences, Muhimbili University of Health and Allied Sciences, Dar es Salaam 65001, Tanzania

**Keywords:** SaferBirths, healthcare workers, mothers, newborns, acceptability

## Abstract

**Background**: SaferBirths Bundle of Care (SBBC) is a package of innovative clinical and training tools coupled with low-dose high-frequency simulation-based on-job training guided by local data. This bundle of care is a new initiative being implemented in 30 health facilities from five regions of Tanzania aiming at improving birth outcomes. **Objective**: To assess the perception of healthcare workers and facility leaders on the “SaferBirths Bundle of Care” towards saving women’s and newborns’ lives at birth. **Method**: We used a qualitative approach using focused group discussion (FGD) and individual interviews. A total of 21 FGD and 43 individual interviews were conducted between August and November 2022. In total, 94 midwives and 12 doctors were involved, some of whom were in leadership roles. The framework method for the analysis of qualitative data was used for analysis. **Results**: Healthcare workers and facility leaders received the bundle well and regarded it as effective in saving lives and improving healthcare provision. Five themes emerged as facilitators to the acceptance of the bundle: (1) the bundle is appropriate to our needs, (2) the training modality and data use fit our context, (3) use of champions and periodic mentorship, (4) learning from our mistakes, and (5) clinical and training tools are of high quality but can be further improved. **Conclusion**: The relevance of SaferBirths Bundle of Care in addressing maternal and perinatal deaths, the quality and modality of training, and the culture that enhances learning from mistakes were among the facilitators of the acceptability of the SBBC. A well-accepted intervention has huge potential for bringing the intended impact in health provision.

## 1. Background

Maternal and perinatal mortality continues to be a health challenge of global concern. Annually, about 295,000 women died during pregnancy and childbirth in 2017, and 5.1 million babies were stillborn or died in their first month of life [1,2]. Almost (98%) all these deaths occurred in low- and middle-income countries [3]. Currently, maternal and perinatal mortality in Tanzania stands at 556 deaths per 100,000 live births and 21 deaths per 100,000 live births, respectively. With these rates, Tanzania is far from achieving the 2030 sustainable development goals (SDGs) targets. Most of these deaths are preventable using low-cost simple interventions [4].

To contribute to this endeavor, Tanzania implemented a proven innovation intervention aimed at reducing maternal and perinatal deaths, the SaferBirths Bundle of Care (SBBC). SBBC is a package of innovative clinical tools (Moyo, NeoBeat, and Upright bag-mask) and innovative training tools (NeoNataliae Live and MamaNatalie) coupled with on-job low-dose and high-frequency simulation-based training guided by local data and feedback loops. Moyo is a tool used in monitoring (intermittently or continuously) fetal heart rate during labor. NeoBeat is a newborn heart meter, which can immediately (within 3 s) detect newborn heart rate upon application [5]. The training cascade is the pivot of SBBC. Fifteen SBBC national facilitators were trained for 13 days, who thereafter supported in training sixty facility-based champions (two from each facility) for 6 days. The national facilitators, assisted by the facility champions, trained healthcare workers at each of the 30 facilities for five days. The facility champions took the lead and motivated healthcare workers (HCWs) in the labor ward to utilize the locally captured clinical data and reports (presented as a dashboard) to identify clinical areas with strengths and weaknesses (debriefing). Periodic debrief meetings (weekly) were introduced to help HCWs reflect and continuously tailor ongoing training and implement improvements in the provision of quality care. Furthermore, there have been periodic supervision and mentorship visits conducted by the national facilitators in collaboration with the regional and district health management teams. These scheduled mentorship and supportive supervision visits have provided an opportunity for two-way communication to continually improve SBBC training practices and experience sharing between mentors/supervisors and mentees/supervisees [6,7]. During these visits, participants have been actively engaged, and there has been an agreement to maintain an environment where participants were neither intimidated nor blamed. The focus has been on the potential improvement of local care, with an appropriate follow-up of gaps identified in the previous mentorship visit. The overall goals of SBBC are to avert maternal deaths associated with postpartum hemorrhage and reduce fresh stillbirths and early neonatal deaths [5].

The overall effectiveness of an intervention depends on the level of acceptability of the intervention. Among the factors that influence users’ perceived acceptability of an intervention include the intervention’s appropriateness in addressing existing clinical problems [8], the content of the intervention, and the perceived or actual effectiveness of the intervention [9]. Low acceptability may result in implementation without fidelity, delivering suboptimal results [10,11]. Acceptability is a term that can be considered from an individual perspective and can collectively reflect shared judgment about the potential of an intervention [9]. We conducted the acceptability study to assess the general HCWs’ and facility leaders’ opinions of the SBBC.

## 2. Methods

### 2.1. Study Design

We employed a qualitative design using individual semi-structured interviews and focus group discussions (FGDs) with HCWs (midwives and doctors) and facility leaders to explore their perceptions and opinions on SBBC. Both individual interviews and FGDs were used to enhance data richness [12].

### 2.2. Study Setting and Timing

The study was conducted in 21 purposely selected facilities in the Manyara, Tabora, Geita, Shinyanga, and Mwanza regions, between August and November 2022. The involved facilities were regional hospitals (four), district hospitals (fifteen), and health centers (two). The facilities were purposely selected to represent the study population. The interviews and FGDs were conducted more than six months after the start of SBBC implementation to make sure that HCWs had enough time to work with the bundle.

### 2.3. Participants and Data Collection

Invitations to participate in the study were extended to midwives and doctors working at the maternity ward and health facility leaders including the medical officer in charge, matron, and labor ward in charge. The HCWs and facility leaders with at least three months of working at the maternity ward and at the leadership position on the day of the interview, respectively, were invited to participate in the FGD or individual interview. Participants varied in age, gender, and number of years of working in the labor ward. Efforts were made to involve facility leaders with at least three months in a leadership position at the facility during the implementation of the SBBC project. This was done to ensure the capturing of a broad spectrum of perspectives of the study objective.

Each interview, both individual and FGD, was conducted using a semi-structured interview guided by two (researcher/research assistant), where one moderated the interview, and the other recorded and took notes. All individual interviews and FGD were conducted in the local language, Kiswahili, by two researchers and two research assistants with a background in public health and obstetrics and health anthropology and midwife tutoring, respectively, with additional training in qualitative research. A total of 21 FGDs and 43 individual interviews were conducted. From each facility, about two individual interviews and one FGD were conducted (Table 1). Some participants from FGDs were selected for additional individual interviews. Facility leaders were voluntarily involved in individual interviews. We did not conduct FGD with facility leaders due to their small number, which did not meet the requirements to be included in FGDs. The interview guides included objective and open-ended questions regarding the SBBC. The areas explored during the interviews were general views of the bundle, level of acceptability, and factors facilitating the acceptability of the bundle. The sense of thematic saturation was felt after several FGD and individual interviews. However, additional FGDs and individual interviews were conducted in an attempt to include more different contexts [13]. All FGDs and individual interviews took place within the facility premises, where privacy was ensured. The FGDs and individual interviews lasted between 30 and 55 min and 25 and 36 min, respectively.

### 2.4. Sampling and Sample Size

A total of 149 HCWs (midwives and doctors) and facility leaders were purposely selected and invited to voluntarily participate in the FGDs and/or individual interviews. All of them accepted. The sample size was based on the data adequacy, richness of the data, and the involvement of a wide range of participants to achieve a wide understanding of the extent to which the bundle is acceptable [14].

### 2.5. Data Management and Analysis

We used the framework method for the analysis of qualitative data [15]. The audio-recorded scripts were transcribed verbatim by an independent transcriber who was not involved in the interviews. The transcripts were then translated from the local language to English by an independent translator and then back-translated to the local language by a different independent translator to verify the retention of the content. Two authors (PM and EM) read and re-read all the transcripts and developed initial codes, sub-themes, and themes. They then shared the initial codes and themes with other authors (BK and RM), who reviewed and refined the themes based on the transcripts. The refined themes were thereafter applied back to raw data to determine the fit and refine as needed. The overall interpretation was confirmed with the inputs from the whole research team to ensure dependability [16].

## 3. Results

The individual interviews and focus group discussions involved HCWs (midwives and doctors) and facility leaders (medical officers in charge (6), nurse officers in charge (8), and labor wards in charge (11)).

The majority (85.2%) of the HCW participants were female. They varied in age, and most of them (74.6%) were between 31 and45 years. More than half of the HCW participants had 1–3 years’ experience working at the maternity ward during the interview. Only 38% of the HCW participants had a bachelor’s or higher education in their field, and the rest were diploma and/or certificate holders. Among the HCWs, midwives were the majority (87.2%), and the rest were doctors (Table 2).

Five themes were identified from both the individual interviews and the FGDs: (1) the bundle is appropriate to our needs, (2) the training modality and data use fit our context, (3) the use of champions and periodic mentorship, (4) learning from our mistakes, and (5) clinical and training tools are of high quality but can be further improved.

### 3.1. Appropriateness of the Bundle to HCW Needs

Participants in both individual interviews and FGDs admitted the appropriateness of the bundle to their existing needs. Participants felt that the tools that came with SBBC have narrowed the gap of staff shortage in their facilities.

“We have a challenge of staff shortage, therefore with this challenge of staff shortage the bundle helps us. For example, in a shift we are two nurses/midwives at that time we have six or ten women in labour. It is not easy to auscultate the fetal heart rate of every woman for every 30 min and others every fifteen minutes using a fetoscope. But now we have Moyo, the fetal heart rate monitoring has become easy for us even with this staff shortage”.

(Midwife, 6 years’ experience)

Furthermore, participants felt that the bundle had helped them to change from the traditional way of providing care to a more modern way.

“The bundle has moved us from the old way of doing things to a new better way of doing things”.

(Hospital matron, 6 years in leadership)

“As we know the primary goal of the SaferBirths Bundle of Care is to reduce maternal and newborn deaths, which has helped us greatly. For example, in the labor ward after training, we have changed our practice. Before the training, we used to suck every newborn even if it has no meconium, but after the training, we were taught to suck newborns with meconium only and not every newborn”.

(Midwife, 8 years’ experience)

Participants acknowledge having the challenge of high numbers of maternal and perinatal deaths in their facilities. Nevertheless, participants confirmed that SBBC had been an appropriate intervention in addressing this challenge.

“We had a challenge with perinatal deaths, we had high numbers of perinatal deaths. When I hear about the bundle, what comes first is that big goal of reducing maternal and newborn deaths”.

(Midwife, 3 years’ experience)

“We all know that pregnancy is not a disease but rather a path for a moment. So, to help people from dying continuously they brought us the training to enable us to be able to save maternal and newborn lives”.

(Doctor, 7 years’ experience)

The participants appreciated the presence of the obvious impact of the bundle on the perinatal outcomes. They said that they had reduced perinatal mortality significantly following the use of the bundle in their facilities.

“We have moved away from the high numbers of perinatal deaths, we used to have 20, 21, 22 perinatal deaths within three months but now, the number has declined significantly, now we have 3, 4 perinatal deaths in three a month time. So, we thank God the bundle has moved us from losing newborns to saving more newborns”.

(Matron, 3 years in leadership)

“I am grateful for the bundle as it has been a catalyst to good services, reduction of deaths and complications that have been occurring”.

(Medical officer in charge, 4 years in leadership)

The midwife in charge of the labor ward went far by admitting the impact of the bundle in reducing postpartum hemorrhage-related maternal deaths in her facility.

“The bundle has helped us to reduce the number of postpartum hemorrhages related maternal deaths, we thank God that now we are saving more women. We have also discovered that as we do more practice/training, it helps us to save those women”.

(Labor ward in charge, 6 years in leadership)

The facility leaders were impressed with the tools in the bundle, and they admitted the fact that the tools had brought a huge impact on the service delivery at their facilities.

“Starting with the maternity ward, the availability of tools that can detect dangers in a pregnant woman and ways to handle them, in that sense it has reduced the number of babies born in bad state such as birth asphyxia. So availability of tools has helped. But also, there are those born floppy, the presence of heart rate alone with NeoBeat has helped. Even those that we could have lost hope on, are saved”.

(Medical officer in charge, 6 years in leadership)

The SBBC project also supported an improvement of the neonatal wards/care unit (NCU). This was appreciated by the facility leaders, who further admitted the impact of this improvement.

“But on the neonatal side, we did not have a neonatal unit, but with SBBC we were enabled to renovate and equip the unit. It has been a great help. Initially, we did not admit neonates even for medication only. Even though we could treat them, but it was in the mixed ward with mothers, the follow-up was very poor, and with the bundle, the unit is functioning as other wards. All these are the fruits of SBBC”.

(District Coordinator of maternal and child health, 9 years in leadership)

Through SBBC implementation, participants admitted to having gained knowledge that subsequently passed over to others who joined the facilities later during SBBC implementation.

“SBBC has helped us, and we have gained knowledge and this knowledge has helped others who are not trained. If untrained staff comes while the trained staff are running a scenario, they gain knowledge from there”.

(Midwife, 4 years’ experience)

### 3.2. The Relevance of Training Modality and the Use of Data 

The participants were pleased with the way the bundle was introduced to them and how the training was conducted. They admitted that the good planning of the training enabled more staff to attend the initial facility training.

“We were first invited to the training for capacitating us to be able to save maternal and newborn lives. ……… SBBC introduction was well planned because most of the staff here were able to attend the training and were able to do practical training using SBBC tools. We had practical training using the SBBC training tools”.

(Midwife, 2 years’ experience)

Participants appreciated the training modality, which started with a theoretical introduction followed by intensive practical sessions. Thereafter, HCWs continued with training on their own at their working places.

“The training lasted for about five days, and all HCWs attended a brief theoretical introduction of the bundle followed with extended practical training sessions”.

(Midwife, 11 years’ experience)

“Apart from the initial training, we continued with training afterward using the training tools we have. Therefore, this continues to enable us to continue providing quality care to our clients”.

(Doctor, 3 years’ experience)

“This training we do ourselves, they help us to improve our experiences and skills and enable us to do better”.

(Midwife, 1 year experience)

Additionally, HCWs perceived that low-dose high-frequency on-job simulation training impacted their skills and confidence in providing better care to patients.

“Frequent self-training helps you to do well when you meet the real situation because you have practiced more frequently. Therefore, it helps you build confidence and better skills to provide the best care to women and newborns”.

(Midwife, 7 years’ experience)

“The low-dose high frequency on-job training has helped us a lot when we meet newborn who needs resuscitation, we are now confident. So, the training has a very positive impact on how we deliver care to our patients. The good thing we see a reduction in newborn deaths at our facility”.

(Doctor, 6 years’ experience)

The use of locally collected data to evaluate the clinical practice was emphasized as very valuable. During local data review and discussions, clinical gaps were identified, and simulation scenario training was planned, aiming to improve clinical skills.

“I think this has helped us a lot, because when we collect and discuss the data, normally there are good and bad things. So, for the positive things, we take them and continue to improve and for the negative ones, maybe we did not do to the baby, we plan for improvement and training so that we continue to save women and their newborns in a timely manner. To improve further we need to match the practical training and the real situation”.

(Midwife, 15 years’ experience)

### 3.3. Engagement of Champions and Periodic Mentorship

Each facility had two champions who were trained and well-equipped in the use of the bundle. These champions were appreciated by their fellow colleagues for being motivators for others to improve the quality of care. Champions planned and facilitated simulated scenario training and communicated the gaps to colleagues in a friendly way.

“It’s been helpful because….it depends, if we have champions, they see to what scenario we should run, let’s say retained placenta, and how we should remove it manually. So, we run the scenario and evaluate where we go wrong so we can rectify it. During debriefing, we learn what we should have done right and the next time we do it’s easier. In the past when we had mothers with retained placenta, we used to call the doctor but now no need. We nurses remove it manually by ourselves”.

(Midwife, 4 years’ experience)

Furthermore, the periodic mentorship conducted by project mentors/national facilitators was mentioned as being among the facilitators for the acceptability of the bundle.

“Supervision is helpful as it helps us know where we have gone wrong and help us correct our mistakes”.

(Midwife, 3 years’ experience)

### 3.4. Learning from Mistakes

Participants appreciated the “no blame culture” that came along with the bundle. They highlighted the growing culture of not blaming staff after encountering adverse outcomes but rather conducting a proper debriefing for learning and improvement.

“Thank you very much, SaferBirths Bundle of Care has one thing which is very good, the “no blaming culture”. It helps a lot for people to be positive because none is blamed, we all train and share experiences, so this helps a lot in the SaferBirths Bundle of Care”.

(Doctor, 2 years’ experience)

“…, it was very scary, once you get say fresh stillbirth, you rush to hide the case note where matron cannot see it, you think what I will say about it, I have done wrong, what will happen to me and so on, so, it was very difficult times. But nowadays if you get fresh stillbirth, you colleagues call you with love, please come let us sit down and discuss the strength and the areas for improvement. We discuss …. identified gaps and make them our objectives for training further that we aim at not repeating the same mistakes tomorrow”.

(Midwife, 5 years’ experience)

They insisted on the fact that people normally learn from mistakes; therefore, a no-blame approach gave them the opportunity to learn and improve their performance.

“You know that people learn from mistakes. If there was a mistake in that maternal death and you want to hide it, the mistake will be done again. But when we do discussions, it helps us know where we have gone wrong and learn so when you have another woman the mistake will not reoccur”.

(Midwife, 7 years’ experience)

### 3.5. The Quality of SBBC Clinical and Training Tools

The tools in the bundle were perceived to be easy to use and have a big impact. Participants had different opinions regarding the tools, and most of them had positive perceptions. NeoBeat (heart meter) was the tool that was mentioned to have impacted perinatal outcomes the most.

“When a woman delivers a floppy baby, we used to check cord pulsation. If it is not there, we cover up the baby and term it dead. But since we got the NeoBeat, even if there is no pulsation of the cord but NeoBeat picks the heart beats, we know the baby is alive and start resuscitation, we have saved most of them by NeoBeat. To a great extent we have saved the lives of neonates that we used to misclassify as dead”.

(Midwife, 9 years’ experience)

Participants admitted the importance of continuous low-dose high-frequency training, which was facilitated by the availability of training tools. The availability and usefulness of the training tools were perceived as useful in improving HCWs’ skills.

“MamaNatalie helps us to practice management of postpartum hemorrhage so when we get a woman with postpartum hemorrhage, we can easily help her. For example, if you have delivered a woman, the third stage, if she continues to bleed may be due to atonic uterus or retained placenta, we should be able to help”.

(Midwife, 6 years’ experience)

Despite accepting the bundle, some participants had opinions about further improving the bundle. The improvement of some tools and the involvement of more health facilities were among the areas of improvement suggested by the participants.

“So, it could have been better if they can improve Moyo to be able to detect two parts of the twins”.

(Midwife, 9 years’ experience)

“Moyo does not record contractions. If we would have been able to know this is strong, moderate, or mild contraction, that could have been of important information”.

(Doctor, 5 years’ experience)

## 4. Discussion

This study found that SBBC was well accepted by both health care workers and facility leaders who were interviewed. Both categories of participants seem to have received the bundle well and regarded it as effective in improving healthcare provision and saving lives. Participants highlighted several things that might have facilitated the acceptance of the bundle: (1) the bundle being appropriate to HCWs’ needs, (2) training modality and data use that fit HCWs’ context, (3) the usefulness of champions and periodic mentorship, (4) the potential for learning from mistakes to improve care provision, and (5) the quality of clinical and training tools.

The perception of acceptability of an intervention can be influenced by learning about the intervention before having engaged with the intervention [17]. This study found that HCWs who were well informed about the bundle accepted it well and were thus motivated when starting to use the bundle. Positive attitudes of HCWs towards the bundle and proper implementation resulted in finding the appropriateness of the bundle to address their needs. The appropriateness of the intervention is a key determinant of its acceptability among users. SBBC is designed to address the burden of maternal and perinatal mortality associated with inadequate quality of health care services at the facilities [5]. The halfway findings of the SBBC implementation have shown a steady increase in 24 h newborn and maternal survival in the five regions [18]. The halfway findings concur with the findings in our study that the SBBC is an appropriate intervention to address the burden of maternal and newborn deaths. The participants admitted changing their ways of managing newborns from old to new ways following the introduction of the SBBC.

The SBBC package targets clinical processes using innovative tools and continuous training to enhance patient outcomes, as has been reported in a systematic review [19]. SBBC employs low-dose high-frequency simulation-based on-job training (LDHF-SBOJT) using simulators. Simulation is the technique for practice and learning that replaces and amplifies real experiences with guided ones [20]. The SBBC training modality seems to have influenced the level of acceptability of the bundle. Knowledge and skills are the foundation for the provision of required care and are supported by training. Simulation training tools are composed of technology (NeoNatalie Live manikin) and manual (MamaNatalie). The SBBC’s innovative tools for training helped HCWs to practice individually or in groups after formulating scenarios derived from collected clinical data [21]. Furthermore, HCWs appreciated the training model for continuous quality improvement supported by innovative tools for continuous skill building and retention over time, which has also been observed in other settings [22]. This study reports the acceptability of LDHF training as a potential aspect of the retention of skills and imparting skills to new staff. Similarly, Mduma et al. (2019) reported retention of skills following LDHF among staff [23]. The uptake of the training modality enhanced clinical care similarly to what Rogers described in the process as “diffusion of innovation”, where the adoption of new innovation enhanced clinical care [24].

Shah has documented that data collection and its use support improvement in care provision [25]. The collection of data and weekly feedback to HCWs helped them to visualize their strengths as motivation and weaknesses. The visualization informed the planning for the following week practicing on the simulators and improvement of care provision. The weekly data sharing was well appreciated, in spite of being a new practice for the health care workers. The findings show that the practice of sharing weekly data with HCWs in the SBBC project has not only made them accept the bundle but also helped them to improve their performance. This has also been documented by Atkinson et al., who found that feedback and coaching help to improve learning, which is important in improving skills [26]. The NeoNatalia Live simulator is used for self-training and practice and gives instant feedback on performance. Practicing accompanied by immediate feedback motivate HCWs to practice when they have free time. Repeated practicing continually builds and retains skills. MamaNatalia manikin is used to practice prevention and management of bleeding after birth that results from the poor contraction of the uterus and retained placenta material after giving birth. The innovative training tools and methodology together with the innovative clinical tools were well accepted by HCWs in all facilities.

Facility-based champions have emerged to be central in facilitating movements for change within the organization or in the process of adopting new interventions [27,28,29,30]. The introduction of facility-based champions during SBBC implementation has been well received and perceived to facilitate the acceptability of the bundle in health facilities. Similarly, Bonawitz K et al. (2020) report that an effective championship appears to leverage influence and ownership at the point of change [31]. Periodic mentorship and supportive supervision were mentioned to further motivate and strengthen skill growth, which is also reported in other studies [32,33].

Not reporting poor birth outcomes before SBBC was reported. Introducing a “no blaming” culture increased transparency and the frequency of reporting poor birth outcomes. The gaps resulting in poor birth outcomes were used to plan training and prevent such outcomes in the future. Solnes Miltenburg et al., in the study conducted between 2014 and 2016 in the Lake Zone of Tanzania, where some of the sites for this study are allocated, found that not reporting adverse outcomes resulted in poor preparation to prevent or accommodate complex labor conditions in the future [34].

Fetal heart rate (FHR) monitoring during labor is a necessary part of labor management for a better newborn outcome. HCWs found that monitoring FHR by using Moyo was easy compared to commonly used Pinard and Doppler, as Moyo required less time. This has also been documented by Mangesi et al. and Mdoe et al. [35,36]. Tools using new technology have been found to be useful in monitoring the progress of fetuses during labor to improve birth outcomes [37]. Similarly, our study found that HCWs were fond of Moyo, as it helped them to monitor FHR effectively.

NeoBeat easily captures newborn heartbeats compared to the commonly used stethoscope. HCWs reported that NeoBeat easily picked up a heart rate, even when the newborn heart rate was low and hard to detect by palpating the umbilicus or auscultating the chest, thus avoiding misclassifying flabby live newborns and stillborns. Such misclassification has been reported in other settings [38].

Using an upright bag mask was found to be easy in ventilating non-breathing newborns and helping them to start breathing. Coffey et al. and Thallinger et al. reported that an upright bag mask was appreciated by HCWs as being more friendly compared to a standard bag mask [39,40]. Despite appreciating the usefulness of the innovative clinical tools, participants in our study suggested further improvement of the tools.

## 5. Limitations

This study informed the perception of HCWs and health leaders towards the SBBC. However, there are several limitations that could challenge our findings. The underrepresentation of participants’ insights may limit our findings, as we only involved facilities leaders and healthcare workers. The inclusion of more diverse HCWs and birthing women, including their partners, may provide more insight. The study did not cover all 30 sites where SBBC was implemented after reaching data saturation. The facilities that were not covered may have a different perception. We collected data at different time points since the initiation of implementation, and thus the perceptions and experience, may change if the study was conducted later, such as at the end of the study. The use of both individual interviews and focus group discussions has enhanced the data triangulation.

## 6. Conclusions

The SBBC project was well accepted by HCWs and facility leaders in the 21 health facilities implementing SBBC. The appropriateness of SBBC in addressing maternal and perinatal deaths, the quality and modality of training, and the culture that enhances learning from mistakes were among the facilitators of the acceptability of the SBBC. A well-accepted intervention has huge potential to bring the intended impact on health provision.

## Figures and Tables

**Table 1:** Number and categories of participants in the FGDs and individual interviews.

	Total Conducted	Total Participants Involved	Categories of Participants
Focus Group Discussions	21	Midwives	94
		Doctors	12
Individual Interviews	43	Medical officer in charge	6
		Nurse officer in charge	14
		Labor ward in charge	14
		HCWs (midwives/doctors)	9

**Table 2:** Characteristics of the HCW participants in the individual interviews and FGDs.

Age Group of HCWs (Years)	
20–30	17 (12.0%)
31–45	106 (74.6%)
>45	19 (13.4%)
Years of experience working in the respective area	
1–3 years	81 (57.0%)
4–5 years	15 (10.5%)
6–10 years	25 (17.7%)
>10 years	21 (14.8%)
Level of education of the HCWs	
Certificate/Diploma	88 (62.0%)
Bachelor and above	54 (38.0%)
Gender of HCWs	
Male	21 (14.8%)
Female	121 (85.2%)
Cadre of the HCWs	
Midwives	124 (87.3%)
Doctors	18 (12.7%)

## Data Availability

Data can be requested from Haydom Lutheran Hospital, through the project managers Paschal Mdoe and Benjamin Kamala.
